# Supplementary material for: Nonlinear relationship between circulating natural killer cell count and 1-year relapse rates in myasthenia gravis: a retrospective cohort study
Source: PeerJ. 2024 Dec 6;12:e18562. doi: 10.7717/peerj.18562 (PMC11627074; doi:10.7717/peerj.18562)
Supplement: Table S1 [file peerj-12-18562-s002.docx]

Supplemental table 1: Antibody Information

| antigen | clone | Fluorochrome |
| --- | --- | --- |
| CD3 | UCHT1 | FITC |
| CD4 | RPA-T4 | PC7 |
| CD8 | HIT8a | APC-Cy7 |
| CD16 | CB16 | PE |
| CD19 | HIB19 | APC |
| CD56 | MEM-188 | PE |
| CD45 | 2D1 | PerCP-Cy5.5 |
