# Supplementary material for: Nonlinear relationship between circulating natural killer cell count and 1-year relapse rates in myasthenia gravis: a retrospective cohort study
Source: PeerJ. 2024 Dec 6;12:e18562. doi: 10.7717/peerj.18562 (PMC11627074; doi:10.7717/peerj.18562)
Supplement: Table S2 [file peerj-12-18562-s003.docx]

Supplemental table 2: The description of missing data

| Variables | 1:Yes | 0:No | Missing rate(%) |
| --- | --- | --- | --- |
| Age | 261 | 4 | 1.51 |
| Gender | 265 | 0 | 0 |
| Thymus | 264 | 1 | 0.38 |
| Osserman classification | 263 | 2 | 0.75 |
| Involvement of respiratory muscles | 265 | 0 | 0 |
| Involvement of limb muscles | 265 | 0 | 0 |
| Involvement of pharyngeal muscles | 265 | 0 | 0 |
| Involvement of extraocular muscles | 265 | 0 | 0 |
| AChR-ab (radioimmunoassay) | 265 | 0 | 0 |
| MUSK-ab | 265 | 0 | 0 |
| Thymectomy | 261 | 4 | 1.51 |
| Acetylcholinesterase inhibitors | 265 | 0 | 0 |
| Steroids | 265 | 0 | 0 |
| Immunosuppressants | 265 | 0 | 0 |
| CD20 rituximab | 265 | 0 | 0 |
