# Supplementary material for: Nonlinear relationship between circulating natural killer cell count and 1-year relapse rates in myasthenia gravis: a retrospective cohort study
Source: PeerJ. 2024 Dec 6;12:e18562. doi: 10.7717/peerj.18562 (PMC11627074; doi:10.7717/peerj.18562)
Supplement: Supplemental Information 4 [file peerj-12-18562-s004.docx]

Supplemental table 3: Variables with a P value less than 0.1 associated with the outcome variable in univariate logistic regression.

| ovariates | exp(beta) | 95%CI Low | 95%CI Upp | P.value |
| --- | --- | --- | --- | --- |
| Gender | 0.8718 | 0.5474 | 1.3885 | 0.5635 |
| Age | 0.9875 | 0.9753 | 0.9998 | 0.0466 |
| Thymus | 1.4845 | 0.9456 | 2.3308 | 0.0860 |
| Thymectomy | 1.4762 | 0.8779 | 2.4822 | 0.1419 |
| AChR-ab (radioimmunoassay) | 1.9062 | 1.1948 | 3.0412 | 0.0068 |
| MUSK-ab | 5.5324 | 1.0566 | 28.9667 | 0.0428 |
| Osserman classification |  |  |  |  |
| II vs I | 1.3157 | 0.8054 | 2.1493 | 0.2733 |
| III vs I | 4.6341 | 2.0316 | 10.5709 | 0.0003 |
| IV vs I | 0.4472 | 0.0959 | 2.0844 | 0.3055 |
| Involvement of limb muscles | 1.7830 | 1.1151 | 2.8509 | 0.0157 |
| Involvement of pharyngeal muscles | 1.8718 | 1.1045 | 3.1722 | 0.0198 |
| Involvement of respiratory muscles | 0.6510 | 0.2324 | 1.8238 | 0.4141 |
| Involvement of extraocular muscles | 2.5801 | 1.5897 | 4.1876 | 0.0001 |
| Involvement of medulla oblongata | 4.3853 | 0.7912 | 24.3060 | 0.0907 |
| Acetylcholinesterase inhibitors | 0.4177 | 0.2605 | 0.6698 | 0.0003 |
| Steroids | 0.4321 | 0.2739 | 0.6817 | 0.0003 |
| Immunosuppressants | 0.9577 | 0.5283 | 1.7361 | 0.8868 |
| Intravenous immunoglobulin (IVIg) | 1.1144 | 0.6087 | 2.0403 | 0.7255 |
| Plasma exchange | inf. | 0.0000 | Inf | 0.9845 |
| CD20 rituximab | 0.5529 | 0.1793 | 1.7054 | 0.3025 |
