# Supplementary material for: Nonlinear relationship between circulating natural killer cell count and 1-year relapse rates in myasthenia gravis: a retrospective cohort study
Source: PeerJ. 2024 Dec 6;12:e18562. doi: 10.7717/peerj.18562 (PMC11627074; doi:10.7717/peerj.18562)
Supplement: Table S4 [file peerj-12-18562-s005.docx]

Supplemental table 4: Covariates impacting the association between Ln(NK cell count) and 1-year relapse-related risk (RR) by more than 10%

| **Covariate** | **Basic Model** | **Full Model** | **Selected** |
| --- | --- | --- | --- |
| Initial Regression Coefficients | -0.7345 | -0.7767 |  |
| Gender | -0.7651 | -0.7402 |  |
| Age | -0.7493 | -0.7024 |  |
| Thymus | -0.7188 | -0.7814 |  |
| Thymectomy | -0.737 | -0.7574 |  |
| AChR-ab | -0.7102 | -0.8172 |  |
| MUSK-ab | -0.8169 * | -0.6918 * | Yes |
| Osserman classification | -0.6570 * | -0.7612 | Yes |
| Involvement of limb muscles | -0.7021 | -0.7298 |  |
| Involvement of pharyngeal muscles | -0.6972 | -0.7494 |  |
| Involvement of respiratory muscles | -0.7544 | -0.7467 |  |
| Involvement of extraocular muscles | -0.698 | -0.8127 |  |
| Involvement of medulla oblongata | -0.7855 | -0.7326 |  |
| Acetylcholinesterase inhibitors | -0.67 | -0.7844 |  |
| Steroids | -0.6757 | -0.7931 |  |
| Immunosuppressants | -0.7469 | -0.7122 |  |
| Intravenous immunoglobulin | -0.7286 | -0.8169 |  |
| Plasma exchange | -0.7208 | -0.7943 |  |
| CD20 rituximab | -0.7408 | -0.7604 |  |
