# Supplementary material for: Nonlinear relationship between circulating natural killer cell count and 1-year relapse rates in myasthenia gravis: a retrospective cohort study
Source: PeerJ. 2024 Dec 6;12:e18562. doi: 10.7717/peerj.18562 (PMC11627074; doi:10.7717/peerj.18562)
Supplement: Table S5 [file peerj-12-18562-s006.docx]

Supplemental table 5: Covariates impacting the association between Ln(NK cell proportion) and 1-year relapse-related risk (RR) by more than 10%

| **Covariate** | **Basic Model** | **Full Model** | **Selected** |
| --- | --- | --- | --- |
| Initial Initial Regression Coefficients | -0.3806 | -0.1548 |  |
| Gender | -0.4062 | -0.1412 |  |
| Age | -0.3270 * | -0.2842 * | Yes |
| Thymus | -0.3522 | -0.1535 |  |
| Thymectomy | -0.3697 | -0.1503 |  |
| AChR-ab | -0.387 | -0.162 |  |
| MUSK-ab | -0.4064 | -0.1414 |  |
| Osserman classification | -0.3477 | -0.1448 |  |
| Involvement of limb muscles | -0.3421 * | -0.1522 | Yes |
| Involvement of pharyngeal muscles | -0.3437 | -0.157 |  |
| Involvement of respiratory muscles | -0.3931 | -0.1279 * | Yes |
| Involvement of extraocular muscles | -0.366 | -0.1699 |  |
| Involvement of medulla oblongata | -0.4088 | -0.1378 * | Yes |
| Acetylcholinesterase inhibitors | -0.2998 * | -0.1776 * | Yes |
| Steroids | -0.3092 * | -0.2135 * | Yes |
| Immunosuppressants | -0.3787 | -0.147 |  |
| Intravenous immunoglobulin | -0.3812 | -0.1813 * | Yes |
| Plasma exchange | -0.3453 | -0.2142 * | Yes |
| CD20 rituximab | -0.4221 * | -0.1255 * | Yes |
