# Supplementary figures and images for: Nonlinear relationship between circulating natural killer cell count and 1-year relapse rates in myasthenia gravis: a retrospective cohort study
Source: PeerJ. 2024 Dec 6;12:e18562. doi: 10.7717/peerj.18562 (PMC11627074; doi:10.7717/peerj.18562)

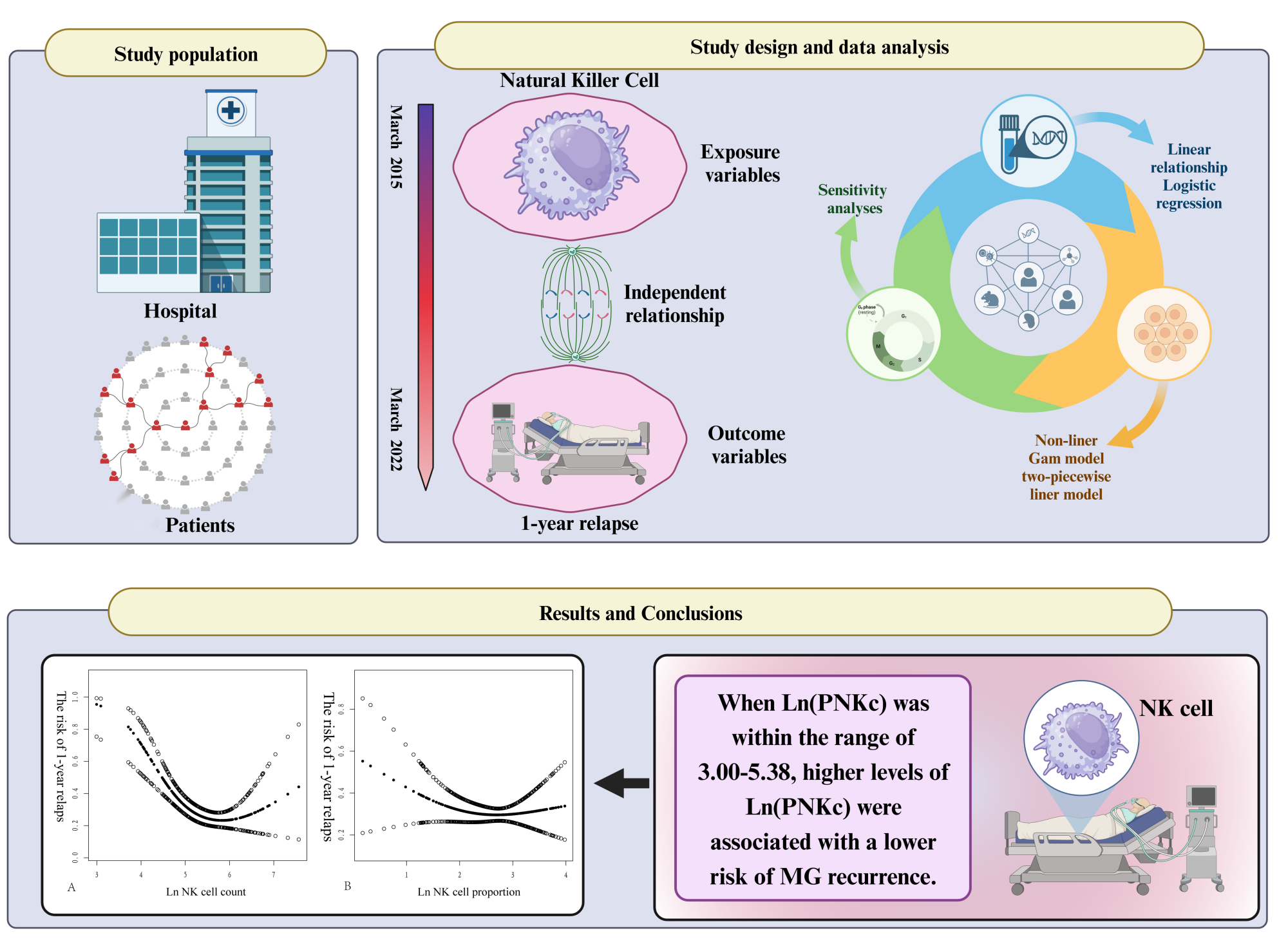

Supplement: Supplemental Information 7 [file peerj-12-18562-s007.png]
